# The resurgence of monkeypox virus: a critical global health challenge and the need for vigilant intervention

**DOI:** 10.3389/fpubh.2025.1572100

**Published:** 2025-05-30

**Authors:** Ashwini Malla, Fayez M. Saleh

**Affiliations:** ^1^Department of Biotechnology, PSG College of Arts and Science, Coimbatore, Tamil Nadu, India; ^2^Department of Medical Microbiology, Faculty of Medicine, University of Tabuk, Tabuk, Saudi Arabia; ^3^Molecular Microbiology and Infectious Diseases Research Unit, University of Tabuk, Tabuk, Saudi Arabia

**Keywords:** global health, monkeypox virus, Orthopoxvirus immunity, outbreak containment, public health strategy, zoonotic diseases

## Abstract

The resurgence of the monkeypox virus (MPXV), a zoonotic Orthopoxvirus historically regarded as endemic to the tropical rainforests of Central and West Africa, represents a significant and evolving global health challenge. Waning Orthopoxvirus immunity following the cessation of smallpox vaccination and inequitable vaccine access have increased susceptibility, especially in resource-limited settings. Combined with urbanization, environmental degradation, global travel, and human-wildlife interactions, these factors have driven MPXV beyond its traditional regions. Notably, recent outbreaks in non-endemic countries have exhibited a distinct epidemiological shift, with a higher incidence among men who have sex with men, often in the absence of travel history to endemic areas, underscoring evolving transmission dynamics. This review provides a comprehensive examination of MPXV’s epidemiology, clinical features, and transmission mechanisms, highlighting the complexities of its containment. Key challenges—including surveillance gaps, vaccine inequities, and limited access to diagnostics and therapeutics—are compounded by unresolved controversies over MPXV’s natural reservoirs and respiratory transmissibility, as well as critical research gaps in zoonotic spillover mechanisms and long-term immunity. Addressing these issues demands global collaboration to leverage next-generation vaccines and antivirals, paired with an integrated public health response: enhanced surveillance, targeted education, and equitable resource allocation. Sustaining these efforts is vital to curbing MPXV’s resurgence and preventing its entrenchment as a global health threat.

## Introduction

Monkeypox virus (MPXV), a zoonotic Orthopoxvirus, has emerged as a significant public health concern in recent decades. Closely related to the Variola virus, the causative agent of smallpox, MPXV is historically considered less virulent and less transmissible. However, the cessation of global smallpox vaccination programs in the late 20th century has inadvertently increased susceptibility to Orthopoxvirus infections in human populations, creating a potential niche for MPXV to expand its epidemiological footprint (1, 2).

Endemic primarily to the tropical rainforest regions of Central and West Africa, MPXV was first identified in 1958 during outbreaks in research monkeys, hence its name. The first human case was documented in the Democratic Republic of the Congo (DRC) in 1970 (3). Historically, MPXV outbreaks were sporadic and localized, linked predominantly to zoonotic transmission through contact with infected animals such as rodents and primates (4). However, changing ecological and social dynamics have facilitated the virus’s transition from an obscure zoonotic pathogen to a re-emerging global threat.

Urbanization, deforestation, and the expansion of human-animal interfaces have significantly increased opportunities for zoonotic spillovers. Additionally, the rise in international travel and interconnected global trade has amplified the risk of MPXV dissemination beyond its endemic regions (5). This shift was evident during recent outbreaks, where clusters of cases were reported in non-endemic countries, including Europe, North America, and Asia, underscoring the virus’s ability to adapt and spread in diverse settings (6). In recognition of the growing global threat posed by MPXV, the World Health Organization (WHO) declared the 2022 monkeypox outbreak a Public Health Emergency of International Concern (PHEIC) on July 23, 2022 (7). Despite concerted public health efforts, containment measures have struggled to curb its spread. Furthermore, atypical clinical manifestations observed in recent cases have complicated contact tracing and epidemiological investigations, highlighting potential shifts in disease presentation and transmission patterns (8).

Clinically, MPXV infection manifests as a febrile illness characterized by a vesiculopustular rash, lymphadenopathy, and systemic symptoms resembling smallpox, albeit with lower case fatality rates (9, 10). The virus is transmitted through direct contact with infected individuals, respiratory droplets, and contaminated fomites, as well as zoonotic pathways (11). Notably, recent outbreaks have highlighted potential shifts in transmission dynamics, including increased human-to-human spread in non-endemic regions, raising concerns about the virus’s epidemiological evolution (12, 13).

Despite its growing significance, several knowledge gaps persist regarding MPXV. These include uncertainties about its reservoir species, factors driving interspecies transmission, and the virus’s capacity for sustained human-to-human transmission (14). Additionally, the re-emergence of MPXV highlights the limitations of existing diagnostic tools and the absence of specific antiviral therapies or broadly accessible vaccines (15). Although the modified vaccinia Ankara (MVA) vaccine and antiviral agents like tecovirimat show promise, their availability and distribution remain limited, particularly in resource-constrained settings (16, 17).

This review provides a comprehensive exploration of MPXV’s zoonotic origins, clinical characteristics, and transmission mechanisms. Furthermore, it delves into the public health challenges posed by the virus, alongside advancements in vaccines, diagnostics, and therapeutics. The discussion also highlights controversies and research priorities, with an emphasis on developing robust surveillance systems and intervention strategies to mitigate future outbreaks.

## Zoonotic origins and transmission dynamics

**Figure 1:**
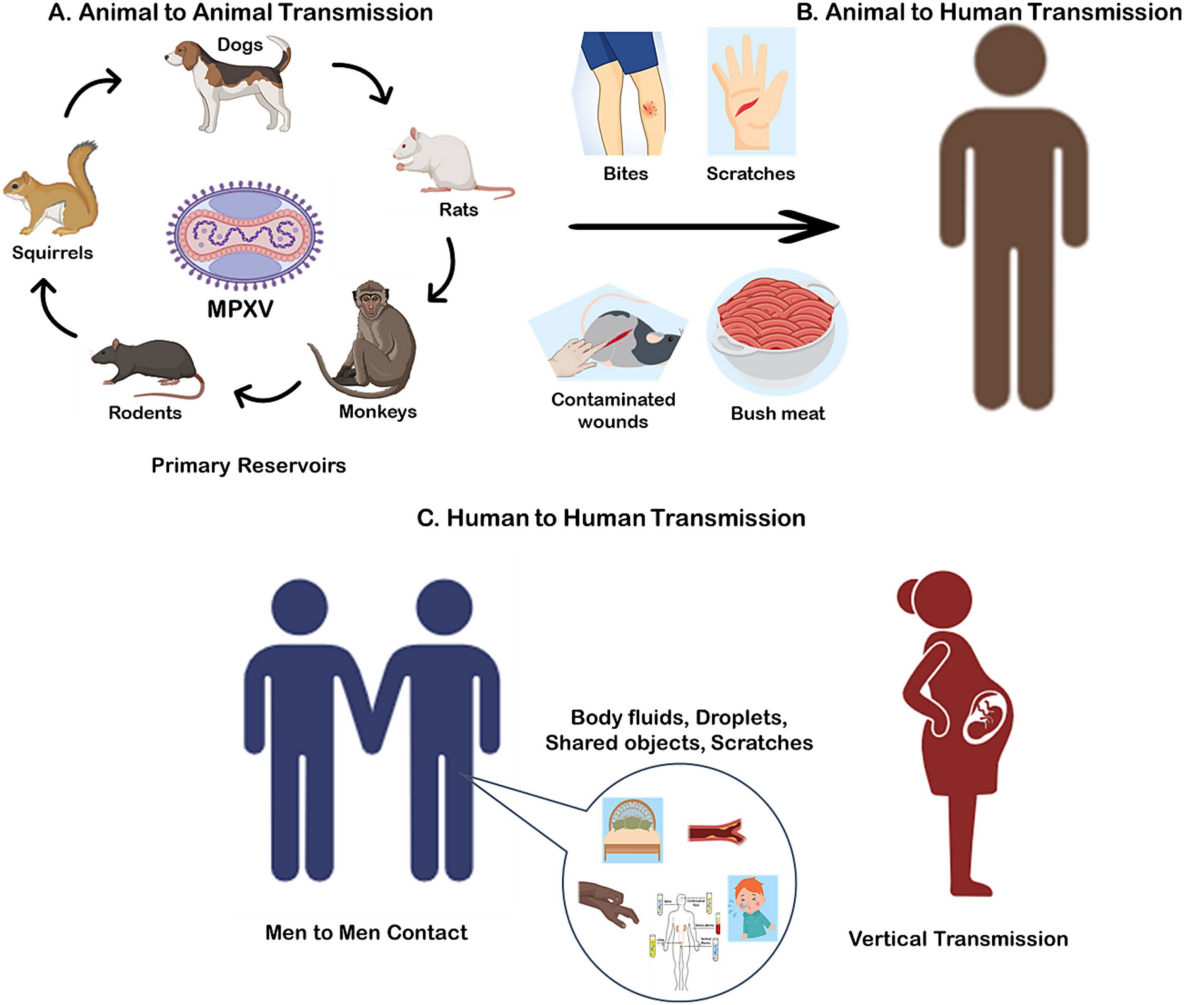
Potential transmission of monkeypox virus (MPXV) via., Animal to Animal **(A)**; Animal to Human **(B)**; and Human to Human **(C)**.

Monkeypox virus (MPXV) is a zoonotic Orthopoxvirus that infects humans through contact with infected animals or via human-to-human transmission. Despite extensive research, the exact natural reservoir of MPXV remains unclear. Small mammals, including rodents such as squirrels (*Funisciurus* spp. and *Heliosciurus* spp.), Gambian pouched rats (*Cricetomys* spp.), and dormice (*Graphiurus* spp.), are considered the primary reservoirs due to their ability to harbor and transmit the virus in endemic regions (18, 19). These animals play a critical role in the virus’s ecological cycle, maintaining its presence in the environment (Figure 1A).

Spillover events from animals to humans are typically associated with direct contact with infected wildlife, such as through hunting, skinning, or consuming bushmeat, which remains a common practice in parts of Central and West Africa (20) (Figure 1B). Environmental degradation, urbanization, and deforestation have amplified these interactions by increasing human encroachment into wildlife habitats, creating conditions conducive to zoonotic spillover (14). In particular, the expansion of agricultural activities and illegal wildlife trade further exacerbate the risk of transmission.

### Human-to-animal transmission

While MPXV is primarily a zoonotic virus, recent reports have highlighted the potential for reverse zoonosis, where infected humans transmit the virus to animals. A notable case in Paris documented the first confirmed instance of human-to-dog transmission, where an Italian greyhound contracted MPXV, likely through close contact with its infected owners (21). This case underscores the importance of isolating infected individuals from pets to prevent potential new animal reservoirs from emerging.

### Human-to-human transmission

Human-to-human transmission of MPXV, although historically less common, has become an increasing concern due to the virus’s ability to exploit densely populated and urbanized environments. Transmission occurs through direct contact with bodily fluids, skin lesions, or contaminated materials such as clothing, bedding, or other fomites (Figure 1C) (22). The changing epidemiology of MPXV highlights its growing adaptability to human hosts. In recent outbreaks, clusters of cases in non-endemic regions have been linked to close interpersonal contact, including sexual networks, emphasizing a potential shift in transmission dynamics (5). Notably, a significant proportion of these cases have been reported among men who have sex with men (MSM), suggesting that intimate contact within this demographic may play a role in the current outbreak’s propagation (23). Additionally, there is evidence to suggest that MPXV can be transmitted via the feto-maternal route, leading to congenital monkeypox (24). This mode of transmission poses significant risks to unborn children, emphasizing the need for heightened awareness and preventive measures among pregnant individuals in affected regions (25).

Genomic surveillance of MPXV isolates from the 2022 outbreaks identified APOBEC3-mediated^1^ mutations in the B.1 lineage, including D209N in the F13L gene (p37 envelope protein) and G2775R in the DNA polymerase gene (26). These mutations are associated with accelerated viral evolution during sustained human-to-human transmission, though their functional impact on transmissibility remains under investigation (27). Unlike earlier outbreaks in rural Africa, where zoonotic spillover was the predominant route, recent data suggest that human-to-human transmission has played a more significant role in propagating outbreaks in urban and global contexts (6). The epidemiology and transmission dynamics of the MPXV have evolved significantly, transitioning from localized, zoonosis-driven outbreaks in endemic regions to sustained human-to-human transmission in global urban settings. Historically, outbreaks in the Democratic Republic of the Congo (DRC) between 2013 and 2015 were characterized by limited human-to-human spread (secondary attack rates of 3–8%), driven primarily by zoonotic spillover from rodents and household clusters in rural communities (28). However, the 2022–2023 global outbreaks marked a paradigm shift, with cases reported in over 100 non-endemic countries, fueled by elevated secondary attack rates (10–15%) and superspreading events linked to high-density gatherings, sexual networks, and international travel (6). This shift reflects the convergence of behavioral factors (e.g., delayed isolation, urban mobility), immunological vulnerability due to waning smallpox vaccine immunity, and globalization enabling rapid cross-border transmission.

### Viral entry and replication

MPXV initiates infection through two primary mechanisms:

#### Direct interaction with host cell receptors

MPXV attaches to host cells via specific interactions between viral surface proteins and cellular receptors (29). Key viral proteins involved in this attachment include D8, which binds to chondroitin sulphate; A27 and H3, which bind to heparan sulphate; and A26, which binds to laminin. Following attachment, the viral envelope fuses with the host cell membrane, leading to the release of viral proteins and enzymes into the cytoplasm. This release facilitates the suppression of host immune defences and initiates the expression of early viral genes (30).

#### Endocytic uptake via macropinocytosis

Alternatively, MPXV can enter host cells through macropinocytosis, an actin dependent endocytic process. In this pathway, the virus is engulfed into vesicles that transport it into the cytoplasm (29).

The entry of MPXV into host cells is mediated by an entry-fusion complex (EFC) composed of eleven proteins. Nine of these proteins (A16, A21, A28, G3, G9, H2, J5, L5, and O3) are integral components of the EFC, while the remaining two (F9 and L1) are associated with the complex. Once inside the host cell, MPXV replicates at the site of inoculation before spreading to regional lymph nodes (22). Unlike many DNA viruses that replicate within the nucleus, MPXV carries the necessary enzymes for transcription and replication, allowing it to replicate entirely within the cytoplasm (31).

**Figure 2:**
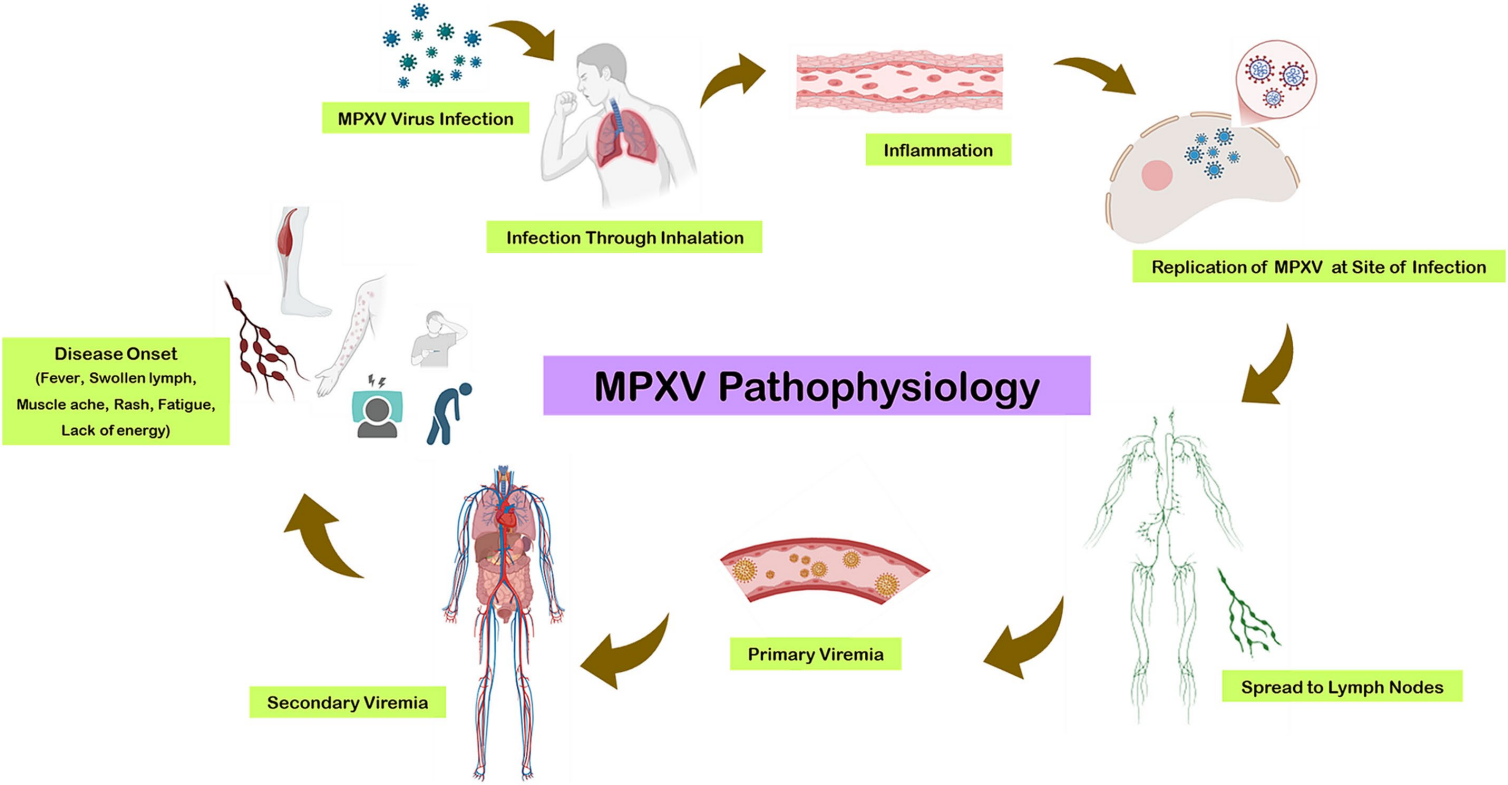
Pathogenesis of monkeypox virus (MPXV).

The replication cycle begins with primary viremia, during which the virus disseminates to various tissues. This incubation period typically lasts from 1 to 2 weeks, extending up to 3 weeks in some cases. Prodromal symptoms, including fever and lymphadenopathy, coincide with secondary viremia, which usually spans 1 to 2 days (Figure 2). During this phase, patients may become infectious. The characteristic lesions of monkeypox first appear in the oropharynx, followed by the development of skin eruptions (22).

The resurgence of MPXV in non-endemic countries underscores the influence of global travel and interconnected human networks in facilitating its spread. International travel during the asymptomatic incubation period has enabled individuals to introduce the virus into new regions, resulting in outbreaks far removed from its endemic zones (2). Moreover, the increasing population of individuals with waning immunity to Orthopoxviruses, due to the cessation of smallpox vaccination campaigns, has created a larger susceptible population, further driving transmission (15). Addressing MPXV transmission requires a multi-pronged approach, including enhanced surveillance, public awareness, and targeted interventions in high-risk areas. Improved understanding of its natural reservoirs, spillover mechanisms, and human-to-human transmission pathways is critical for developing effective containment strategies and minimizing future outbreaks.

### Differential diagnosis of MPXV

The differential diagnosis of monkeypox necessitates distinguishing it from clinically similar exanthematous illnesses (Table 1). Key considerations include varicella (chickenpox), smallpox, measles, herpes zoster, secondary syphilis, herpes simplex virus (HSV) infection, and bacterial folliculitis (32, 33). Monkeypox’s prodromal phase—fever, lymphadenopathy, and myalgia—overlaps with smallpox, but lymphadenopathy (present in 50–90% of monkeypox cases) is a critical differentiator, as it is rare in smallpox and varicella (34). Varicella lesions, unlike monkeypox’s synchronous, deep-seated, umbilicated pustules, evolve asynchronously and are superficial, with centripetal distribution (35). Measles presents with Koplik spots and a cephalocaudal maculopapular rash, accompanied by respiratory symptoms (e.g., cough, coryza), absent in monkeypox (35, 36). Herpes zoster manifests unilaterally along dermatomes, with neuropathic pain preceding the rash (37). Secondary syphilis may involve palmar/plantar papules, with positive serology (RPR/VDRL, FTA-ABS) (38). Herpes simplex virus lesions are mucosal-focused, with PCR confirmation critical (39). Diagnostically, PCR of vesicular fluid (targeting Orthopoxvirus DNA polymerase) is definitive for monkeypox, while electron microscopy identifies brick-shaped virions (40). Epidemiologic context—contact with rodents, endemic travel, or sexual networks—further aids differentiation.

**Table 1:** Key differences among monkeypox and other rash illnesses, emphasizing lymph node findings, rash pattern, and lesion stages.

Feature	Monkeypox (Mpox)	Smallpox	Chickenpox (varicella)	Measles	Secondary Syphilis	HSV (genital)
Prodrome/symptoms	Fever, headache, myalgia 1–4 days *before* rash	High fever, malaise 2–4 days before rash	Mild fever, malaise; rash appears with or after low fever	High fever, cough, coryza, conjunctivitis; *Koplik spots* on buccal mucosa	Systemic symptoms (fever, fatigue) often with rash (weeks after chancre)	Local prodrome: tingling/pain at site; fever/malaise possible (primary infection)
Lymphadenopathy	Prominent (cervical, axillary, inguinal lymph nodes)	Typically, absent or mild	Usually none or mild	Mild (post-auricular, occipital)	Generalized; non-tender (often present)	Tender local nodes (e.g., inguinal)
Rash distribution	Centrifugal: face/extremities (including palms/soles); 2022 cases often perigenital	Centrifugal: face, arms, legs (palms/soles common)	Centripetal: trunk > > face; extremities; includes scalp; lesions at all stages	Begins on face/head → spreads downward; maculopapular (no vesicles)	Generalized (incl. Palms/soles); may involve mucous membranes (condyloma lata)	Localized to genitals, perianal, or oral; grouped blisters/ulcers on erythematous base
Lesion stages	Generally *monomorphic* in classic cases (all same stage in an area); 2022 outbreak often *pleomorphic*	Monomorphic: all lesions same stage (synchronous)	Polymorphic: lesions appear in crops (macules, vesicles, crusts simultaneously)	N/A (no vesicular rash; sequential rash coloration)	Non-vesicular; maculopapular/papulosquamous rash (no uniform “stage”)	Clusters of vesicles; new crops may form over days (semi-asynchronous)
Other clues	Lesions deep-seated, often painful; may have mucosal involvement (oral/genital)	Deep pustules; high mortality (~30%) if variola major	Superficial, pruritic “dew-drop on rose” vesicles; usually milder			

## Clinical features and disease severity

Monkeypox virus (MPXV) infection presents a range of clinical manifestations, from mild self-limiting symptoms to severe systemic complications, depending on the host’s immune status and comorbidities. Understanding these clinical features is crucial for timely diagnosis, effective management, and public health interventions to mitigate the disease burden.

### Typical presentation

The clinical course of MPXV infection begins with a prodromal phase characterized by nonspecific symptoms such as fever, headache, myalgia, fatigue, and chills. Lymphadenopathy is a distinctive feature of MPXV and serves as a critical diagnostic hallmark, differentiating it from other pox-like illnesses, such as smallpox and varicella (10). Swelling of lymph nodes is typically localized to cervical, axillary, or inguinal regions and appears early in the disease course.

Within 1 to 3 days of the prodromal phase, a vesiculopustular rash emerges, initially affecting the face before spreading to other parts of the body, including the palms and soles (41). Lesions progress synchronously through stages of macules, papules, vesicles, pustules, and crusts. The rash is often painful and itchy, with the number of lesions varying widely between individuals. In some cases, mucosal involvement, including oral and genital lesions, has been reported, complicating patient comfort and disease management (4).

The incubation period for MPXV ranges from 5 to 21 days, with symptoms typically resolving within 2 to 4 weeks. However, the severity of clinical manifestations can vary significantly depending on the strain of the virus, with the Central African (Congo Basin) clade historically associated with higher mortality rates and more severe disease compared to the West African clade (1). Recent systematic reviews report an overall Mpox case fatality rate (CFR) of ~8.7%, with pronounced differences by MPXV clade. Central African (Congo Basin, “Clade I”) viruses have a CFR of ~10.6%, whereas West African (“Clade II,” including the predominant 2022–23 outbreak clade IIb) viruses have a CFR around 3.6% (1).

Quantitative analysis of MPXV viral loads across transmission routes reveals stark contrasts in viral shedding. Skin lesions exhibit the highest viral loads, as measured by RT-PCR in vesicular fluid from 2022 outbreak cases (42, 43). Oropharyngeal swabs show moderate shedding, while blood and semen samples demonstrate lower but detectable levels, respectively, (44, 45). Aerosol studies in clinical settings detected MPXV DNA in air samples near infected patients, though culturable virus was rare, suggesting limited infectious potential under natural conditions (46). MPXV respiratory droplets generated during prolonged face-to-face interactions also serve as a mode of transmission, though this is less efficient compared to direct physical contact. In contrast, respiratory droplet transmission appears contingent on prolonged exposure to individuals with high oropharyngeal loads (46). MPXV’s environmental persistence is influenced by temperature and humidity, with longer survival on surfaces under cool, moist conditions. Aerosolized virus likely degrades quickly, favoring direct contact as the primary transmission route (47, 48).

### Severe complications

While the majority of MPXV cases are self-limiting, severe complications can arise, particularly among vulnerable populations. These include young children, pregnant women, the older adult, and immunocompromised individuals, who are at increased risk of systemic involvement. Common complications include:

1. **Encephalitis:** Neurological complications, such as encephalitis, have been reported in severe cases, leading to altered mental status, seizures, and long-term neurological sequelae. These complications highlight the importance of early recognition and supportive care for patients with central nervous system involvement (15).
2. **Sepsis and Secondary Bacterial Infections:** The breakdown of the skin barrier due to MPXV lesions predisposes individuals to secondary bacterial infections, which can escalate to sepsis if untreated. Complications such as cellulitis, abscess formation, and pneumonia have been noted in immunocompromised patients (49).
3. **Ocular Involvement:** In some cases, MPXV has been associated with keratitis, conjunctivitis, and corneal ulceration, which, if left untreated, may lead to vision impairment or blindness (50).
4. **Pregnancy-Related Risks:** Pregnant women infected with MPXV face unique risks, including vertical transmission to the foetus, leading to adverse outcomes such as miscarriage, stillbirth, or congenital monkeypox (51).
5. **Respiratory Complications:** Emerging evidence suggests that respiratory transmission of MPXV, although less common, poses an underappreciated risk in healthcare settings. Patients may develop respiratory symptoms such as cough, sore throat, and bronchopneumonia in severe cases, particularly during prolonged hospital stays (46).

### Host factors influencing disease severity

The clinical severity of MPXV is influenced by various host factors, including age, immune status, and prior vaccination history. Younger populations born after the cessation of smallpox vaccination programs are at heightened risk of severe disease due to the absence of cross-protective immunity provided by the smallpox vaccine (12). Additionally, individuals with immunosuppressive conditions, such as HIV/AIDS or those undergoing chemotherapy, have shown a higher propensity for severe manifestations, prolonged viral shedding, and delayed recovery (1). Host factors also strongly modulate mortality. For example, a study of 57 patients hospitalized with severe Mpox during the 2022 outbreak found that 90% were immunocompromised (primarily advanced HIV), and five deaths occurred (≈21% fatality in that group) (52). These data underscore that immunosuppressed patients (and by extension, others with significant comorbidities) have much higher risk of severe outcomes.

### Vulnerable populations: paediatric and immunocompromised hosts

Contemporary data from non-endemic regions during the 2022 global outbreak (clade II) suggest that paediatric Mpox cases remain rare and are associated with favourable clinical outcomes. A U.S. Center for Disease Control and Prevention (CDC) surveillance report documented that individuals under 18 years of age constituted only 0.3% of confirmed Mpox cases between May and September 2022, with no reported paediatric intensive care admissions or fatalities (53). These findings underscore the relatively low morbidity of clade II Mpox in paediatric populations during recent outbreaks. However, historical evidence from endemic regions highlights divergent risks associated with clade I Mpox. Surveillance data from the Democratic Republic of the Congo (DRC) during 1980–1985 revealed markedly higher case fatality rates (CFR: ~15%) among children aged ≤4 years (3, 54). This disparity in outcomes, further contextualized by a systematic review of Mpox epidemiology (1), emphasizes the dual influence of viral clade and host age on disease severity. While clade II outbreaks have not yet replicated the high paediatric mortality observed in clade I endemic settings, these data reinforce the need for vigilant clinical monitoring in young children, particularly in regions with active clade I transmission or potential clade-specific shifts in viral epidemiology.

Emerging evidence suggests that non-HIV immunocompromised populations—including solid-organ transplant recipients and individuals receiving chemotherapy—face elevated risks of severe Mpox outcomes, though data remain limited. A multicenter U.S. case series reported 11 Mpox infections among solid-organ transplant recipients undergoing active immunosuppressive therapy, with one fatal case attributed to Mpox complications (55). Similarly, a systematic review of 12 documented Mpox cases in kidney transplant recipients identified two fatalities: one due to encephalopathy and multiorgan failure, and another secondary to pneumonitis and septic shock (56). These observations parallel the morbidity patterns seen in advanced HIV-associated immunosuppression, underscoring that iatrogenic or malignancy-related immune dysfunction may similarly predispose patients to severe or fatal Mpox. While current evidence is derived from small cohorts, the reported case fatality rates (9–17% in these groups) emphasize the need for heightened clinical suspicion, early antiviral intervention, and tailored prevention strategies in immunocompromised populations (55, 56).

### Clinical variability in non-endemic outbreaks

Recent outbreaks in non-endemic regions have exhibited variations in clinical presentation compared to historical reports. Notably, patients in these outbreaks have presented with atypical features, such as a localized or sparse rash predominantly affecting the genital, perigenital and perianal regions, often in the absence of systemic symptoms (43). Additionally, some patients have exhibited mild or absent prodromal symptoms, including fever and lymphadenopathy, which traditionally precede the rash onset (30). Such presentations, particularly among individuals engaged in high-risk behaviors, have complicated early detection and contact tracing efforts.

### Implications for healthcare settings

The increasing recognition of MPXV as a global health threat underscores the need for heightened vigilance among healthcare professionals. The potential for nosocomial transmission through contact with contaminated materials or respiratory droplets in poorly ventilated spaces emphasizes the importance of implementing infection prevention and control measures. This includes the use of personal protective equipment, isolation of suspected cases, and rigorous decontamination protocols in healthcare facilities (57).

### Challenges in containment

The global re-emergence and spread of monkeypox virus (MPXV) to non-endemic regions underscore critical gaps in public health infrastructure and highlight systemic challenges in containment. Addressing these challenges is essential for preventing further outbreaks, minimizing morbidity and mortality, and bolstering pandemic preparedness.

### Global mobility and surveillance gaps

The increasing interconnectedness of the modern world, driven by globalization and international travel, has facilitated the rapid spread of MPXV beyond its historically endemic regions in Central and West Africa. Unlike earlier outbreaks, recent cases have been reported in multiple non-endemic regions, including Europe, North America, and Asia, highlighting weaknesses in global surveillance systems and response mechanisms (58).

A major barrier to effective containment is the lack of familiarity with MPXV among healthcare providers in non-endemic regions. Misdiagnosis or delayed recognition of clinical symptoms, such as vesiculopustular rashes, has often resulted in delayed isolation and treatment of infected individuals, inadvertently allowing further community transmission (1). Additionally, atypical presentations observed during recent outbreaks, such as localized genital lesions without systemic symptoms, have complicated early detection, particularly in high-risk populations such as men who have sex with men (5).

Surveillance gaps are further exacerbated by underreporting and the lack of robust diagnostic capacity in resource-limited settings. In many endemic regions, healthcare systems lack the infrastructure, trained personnel, and laboratory resources needed to accurately detect and monitor cases. This issue is compounded by stigma surrounding MPXV infections, which discourages individuals from seeking medical attention, leading to unrecognized and unreported cases (59).

### Waning immunity and vaccine inequities

The cessation of global smallpox vaccination programs in the late 20th century has inadvertently created an “immunological void,” leaving populations increasingly susceptible to Orthopoxviruses like MPXV. Historical evidence indicates that smallpox vaccination confers ~85% cross-protection against severe monkeypox, but waning immunity among vaccinated cohorts and the absence of vaccination in younger generations have amplified vulnerability globally (1, 15). This vulnerability is compounded by stark inequities in access to newer vaccines, such as the Modified Vaccinia Ankara (MVA-BN) vaccine, which has demonstrated 85–90% efficacy against MPXV in clinical trials (60).

Despite their promise, vaccine distribution remains heavily skewed toward high-income countries, leaving endemic regions in Central and West Africa with limited access. Logistical challenges—including inadequate cold-chain infrastructure, insufficient healthcare workforce, and economic barriers—further hinder immunization campaigns in resource-limited settings (61, 62). For instance, during the 2022–2023 outbreaks, non-endemic countries rapidly mobilized vaccine stockpiles, while endemic regions struggled to secure even basic supplies. These inequities perpetuate cycles of vulnerability, enabling MPXV to exploit immunologically naïve populations and facilitating cross-border spread.

Addressing this dual challenge requires reconciling historical policy gaps with modern equity imperatives. Revitalizing vaccination programs in endemic regions, coupled with global mechanisms for equitable resource allocation (e.g., the WHO’s ACT Accelerator model), is critical to curbing MPXV’s resurgence.

### Diagnostic and therapeutic gaps

Accurate and timely diagnosis is critical for breaking the chain of transmission. However, access to advanced diagnostic tools, such as polymerase chain reaction (PCR) assays, remains limited in many endemic regions. These diagnostic delays not only hinder case detection but also prevent effective contact tracing and isolation measures. In non-endemic regions, the sudden demand for diagnostics during outbreaks has overwhelmed laboratory capacity, further delaying diagnosis and public health responses (63).

Therapeutic options for MPXV are also limited. Although antiviral agents like tecovirimat (TPOXX) and brincidofovir have shown promise in treating severe cases, their availability is restricted, and clinical data on their efficacy in real-world settings remain sparse (64, 65). Endemic countries, in particular, face significant barriers to accessing these therapeutics due to high costs, limited distribution channels, and regulatory hurdles.

### Stigma and misinformation

Stigma surrounding MPXV infections, particularly in the context of recent outbreaks affecting marginalized communities, poses another challenge to containment. Misinformation about the transmission of MPXV has led to fear and discrimination, discouraging affected individuals from seeking medical care or disclosing their contacts, thereby hindering public health efforts (5). Public health campaigns aimed at raising awareness and reducing stigma are critical to encouraging timely healthcare-seeking behaviors and ensuring equitable access to testing and treatment.

### Environmental and socioeconomic factors

Environmental and socioeconomic factors also play a significant role in the spread of MPXV. Deforestation, urbanization, and changing land use patterns have increased human-wildlife interactions, raising the risk of zoonotic spillover events (66). Bushmeat consumption, a common practice in endemic regions, serves as a significant driver of MPXV transmission. Efforts to educate communities about the risks associated with bushmeat, combined with initiatives to provide alternative livelihoods, are essential for reducing spillover risks.

Socioeconomic disparities further exacerbate containment challenges. Populations in endemic regions often face limited access to healthcare, poor sanitation, and crowded living conditions, all of which facilitate the spread of infectious diseases like MPXV. Addressing these systemic issues requires a multifaceted approach that combines investments in healthcare infrastructure, community engagement, and poverty alleviation.

### Global coordination and preparedness

The challenges associated with MPXV containment highlight the need for a coordinated global response. International collaboration is essential for addressing vaccine inequities, enhancing diagnostic capacity, and conducting research on MPXV epidemiology, clinical features, and interventions. Danladi et al. (61) emphasize that urban outbreaks require tailored strategies, such as leveraging digital tools for vaccine distribution logistics, while rural areas need community engagement to combat misinformation. Organizations such as the World Health Organization (WHO) and the Coalition for Epidemic Preparedness Innovations (CEPI) play a critical role in fostering global solidarity and ensuring equitable access to resources (1).

Strengthening surveillance systems, particularly in endemic regions, is a priority for improving early detection and response to MPXV outbreaks. Integrating MPXV surveillance into existing zoonotic disease monitoring programs, leveraging digital health tools, and fostering partnerships between public health agencies and academic institutions can enhance global preparedness for future outbreaks.

### Controversies and research gaps

The global resurgence of monkeypox virus (MPXV) has brought attention to critical unresolved scientific and epidemiological questions. Addressing these controversies and research gaps is crucial for improving the understanding of MPXV’s biology, transmission, and public health impact. This section delves into three key areas of ongoing debate and highlights the need for further research to inform effective prevention and containment strategies.

### Unresolved questions on zoonotic reservoirs

One of the most persistent gaps in MPXV research is the identification of its natural reservoirs. While small mammals, particularly rodents, are strongly suspected to act as primary reservoirs, the exact species remain unidentified (18). The uncertainty surrounding the zoonotic origins complicates efforts to predict and prevent spillover events. Without definitive knowledge of reservoir species, surveillance and intervention programs cannot be effectively targeted, increasing the risk of undetected transmission cycles in wildlife populations.

Environmental changes, such as deforestation, agricultural expansion, and urbanization, exacerbate the risks of human-wildlife interactions that could lead to zoonotic spillover. These activities disrupt natural ecosystems and bring humans into closer contact with potential reservoir species (66). For example, bushmeat hunting and consumption are common in endemic regions of Central and West Africa, further facilitating the transmission of MPXV from wildlife to humans. However, the absence of precise data on reservoir species limits the development of targeted policies to mitigate these risks.

Current research is focused on genomic and serological studies to identify potential reservoir species, but challenges remain due to the complexity of MPXV’s ecology and its ability to infect a wide range of hosts. Studies utilizing next-generation sequencing and ecological modeling are needed to uncover the dynamics of MPXV transmission in wildlife populations and their spillover into human communities.

### Potential for respiratory transmission

The role of respiratory transmission in MPXV outbreaks remains a contentious issue in the scientific community. While direct contact with infected bodily fluids or lesions is the primary mode of transmission, there is evidence that respiratory droplets may facilitate person-to-person spread, particularly during prolonged close contact (46). This potential route of transmission raises concerns about the virus’s ability to spread in densely populated urban settings or healthcare environments where prolonged exposure to infected individuals is more likely.

The extent to which respiratory transmission contributes to outbreak dynamics remains unclear. Studies have shown that respiratory transmission is possible in experimental animal models, but its epidemiological significance in human outbreaks has not been definitively established (67). This lack of clarity has led to debates about the adequacy of current infection control measures, particularly in non-endemic regions where healthcare systems may be unprepared for respiratory-transmitted pathogens.

Further research is needed to elucidate the mechanisms underlying respiratory transmission, including the potential for aerosolized particles to carry infectious virus and the environmental conditions that facilitate this mode of spread. Understanding these factors is critical for informing infection prevention protocols, especially in high-risk settings such as hospitals and long-term care facilities.

### Controversies in genomic variability and virulence

Another area of debate centers around the genetic evolution of MPXV and its implications for virulence and transmissibility. Genomic analyses of MPXV isolates from recent outbreaks in non-endemic regions have revealed mutations that may influence the virus’s ability to adapt to human hosts (26). However, the functional significance of these mutations remains poorly understood, and their role in driving outbreaks is a subject of ongoing research.

Some experts speculate that the increased frequency of human-to-human transmission observed in recent outbreaks could be attributed to viral adaptations, while others argue that changes in human behavior and environmental factors are more likely explanations. Resolving these questions will require a multidisciplinary approach, combining virology, epidemiology, and computational modeling to assess the interplay between viral genetics and transmission dynamics.

### Potential developments and future directions

The global resurgence of monkeypox virus (MPXV) has necessitated the exploration of innovative approaches for prevention, diagnosis, and treatment. With advancements in vaccines, antivirals, and public health strategies, there is potential for effective control of MPXV outbreaks. However, these efforts must overcome significant logistical, scientific, and equity-related challenges. This section explores potential developments and future directions for MPXV management, focusing on vaccines, therapeutics, public health strategies, and international collaboration.

### Advancements in vaccines and therapeutics

The Modified Vaccinia Ankara (MVA-BN) vaccine and antivirals like Tecovirimat (ST-246) have emerged as promising tools for managing MPXV infections. The MVA-BN vaccine, a third-generation smallpox vaccine, has shown efficacy in providing cross-protection against MPXV due to its ability to induce robust humoral and cellular immunity (19). Unlike earlier vaccines, MVA-BN is non-replicating, reducing the risk of adverse effects and making it suitable for immunocompromised individuals. However, its effectiveness during outbreaks in non-endemic regions is yet to be fully evaluated through large-scale studies. The MVA-BN vaccine demonstrates 85–90% efficacy in preventing symptomatic monkeypox virus infection, with robust immunogenicity and cross-protective neutralizing antibody responses observed in clinical trials and real-world outbreak settings (60).

Tecovirimat, an FDA-approved antiviral for smallpox, inhibits the viral envelope protein involved in extracellular virion formation, preventing viral spread within the host (68). Initial studies suggest that Tecovirimat may also be effective against MPXV, particularly in reducing disease severity and mortality. Emerging tecovirimat resistance has been linked to mutations in the F13L gene observed in <1% of sequenced cases in 2022–2023 (69). Additional antivirals under investigation, such as brincidofovir and cidofovir, have shown potential efficacy, although their clinical use is often limited by side effects, such as nephrotoxicity (65). The development of targeted antivirals for MPXV remains a high priority, as current treatments are not universally effective.

One of the major challenges lies in ensuring equitable access to these medical interventions. High production costs, limited manufacturing capacity, and logistical hurdles in distribution restrict access, particularly in endemic regions of Central and West Africa. Addressing these challenges will require concerted efforts to scale up production and establish fair allocation mechanisms, especially during global outbreaks.

### Innovations in diagnostic tools

Timely and accurate diagnosis is critical for effective MPXV containment. Traditional diagnostic methods, such as polymerase chain reaction (PCR), remain the gold standard for MPXV detection due to their high specificity and sensitivity. However, the reliance on centralized laboratories for PCR testing can delay diagnosis in resource-limited settings (70, 71).

Recent advancements in point-of-care diagnostic technologies offer promise for rapid and decentralized testing. Isothermal amplification techniques, such as loop-mediated isothermal amplification (LAMP), are being explored for MPXV detection and have shown potential as rapid, cost-effective alternatives to PCR (72). Additionally, immunoassays, including lateral flow assays for antigen or antibody detection, are under development to provide real-time diagnostic capabilities in field settings.

Another area of innovation lies in metagenomic sequencing, which allows for comprehensive detection of pathogens in clinical and environmental samples. While currently limited to research settings due to high costs and technical complexity, advancements in portable sequencing platforms could make this technology more accessible in the future. Improving diagnostic infrastructure and accessibility in endemic regions is essential for early detection and outbreak control.

### Strengthening public health strategies

Public health strategies remain central to MPXV prevention and containment. Educating communities about zoonotic risks, including safe handling of animals and bushmeat, is critical for reducing spillover events. Awareness campaigns emphasizing hygiene practices and early recognition of MPXV symptoms can empower communities to seek timely medical attention, limiting transmission (66).

Enhanced surveillance systems are vital for tracking MPXV outbreaks and identifying high-risk areas. Integrating MPXV surveillance into existing disease monitoring frameworks, such as those for zoonotic diseases or emerging infectious diseases, can improve the efficiency of data collection and analysis. The use of digital tools, including mobile applications and geographic information systems (GIS), can enhance outbreak tracking and resource allocation (73).

Immunization campaigns targeting high-risk populations, such as healthcare workers and close contacts of confirmed cases, can play a pivotal role in outbreak containment. The implementation of ring vaccination strategies, which involve vaccinating individuals within a defined radius of confirmed cases, has shown promise in controlling localized outbreaks. However, the success of such campaigns depends on vaccine availability and public willingness to participate.

### Addressing global inequities

One of the most significant barriers to effective MPXV management is the stark inequity in access to vaccines, diagnostics, and therapeutics. While non-endemic countries have rapidly mobilized resources to respond to outbreaks, endemic regions continue to face shortages of essential medical supplies and infrastructure. For example, endemic countries often lack sufficient vaccine stocks or the cold-chain systems required for vaccine storage and distribution (62).

Efforts to address these disparities must prioritize investment in healthcare infrastructure, workforce training, and resource allocation in endemic regions. Global health organizations, such as the World Health Organization (WHO), can play a key role in coordinating international efforts to ensure equitable access. Initiatives like the WHO’s Access to COVID-19 Tools (ACT) Accelerator provide a model for establishing global mechanisms to facilitate the distribution of MPXV-related interventions.

### International collaboration for research and policy

The global nature of recent MPXV outbreaks underscores the need for coordinated international collaboration in research, policy development, and outbreak response. Collaborative research efforts can accelerate the development of new vaccines, therapeutics, and diagnostic tools while addressing knowledge gaps in MPXV ecology, transmission, and epidemiology. Multinational consortia, such as the Coalition for Epidemic Preparedness Innovations (CEPI), have demonstrated the potential of joint research initiatives in tackling emerging infectious diseases (74).

Policy harmonization is another critical aspect of international collaboration. Standardizing guidelines for surveillance, diagnosis, and treatment can facilitate a unified global response to MPXV outbreaks. Cross-border cooperation in outbreak preparedness, including the establishment of regional response teams and resource-sharing agreements, can enhance the capacity of countries to respond to emerging threats.

### Future directions

Looking forward, the integration of advanced technologies into MPXV management offers exciting possibilities. Artificial intelligence and machine learning could revolutionize outbreak prediction and resource optimization by analyzing large datasets to identify trends and allocate resources effectively (75). Additionally, advancements in synthetic biology and genetic engineering may pave the way for the development of more targeted vaccines and antivirals.

Ring vaccination, which involves immunizing close contacts of confirmed cases and high-risk individuals, effectively limits MPXV spread by disrupting transmission chains. This strategy reduced secondary attack rates by 40–60% during the 2022 global outbreaks and curtailed outbreak duration in regions like Nigeria, where targeted immunization prioritized zoonotic spillover hotspots and dense urban networks (13, 58, 76, 77). Mandja et al. (66) highlight that community engagement and ecological surveillance are critical for identifying zoonotic spillover risks in rural DRC, but they do not provide statistical metrics on case detection rates.

Efforts to combat MPXV must also consider the broader implications of emerging infectious diseases, particularly in the context of climate change and biodiversity loss. Strengthening global One Health initiatives, which recognize the interconnectedness of human, animal, and environmental health, will be crucial for addressing the root causes of zoonotic spillovers and preventing future outbreaks.

## Discussion

The resurgence of monkeypox virus (MPXV) signifies the intricate interplay of ecological, social, and immunological factors that shape its epidemiology. Recent outbreaks in non-endemic regions have exposed vulnerabilities in global health systems, emphasizing the urgent need for integrated, multidisciplinary responses. While advancements in virology and clinical understanding have enhanced our ability to manage MPXV, critical knowledge gaps and challenges remain unresolved. Addressing these gaps will require collaboration between public health, virology, immunology, and social sciences.

One of the most pressing gaps lies in understanding the natural reservoirs of MPXV. Despite extensive research, the primary animal hosts remain unidentified, which hinders efforts to predict and mitigate spillover events (28). This uncertainty complicates the development of targeted interventions to reduce human exposure. Moreover, the ongoing controversy surrounding respiratory transmission of MPXV highlights the need for focused research to elucidate its role in sustaining outbreaks. Determining the relative contribution of respiratory transmission compared to contact transmission could significantly influence public health recommendations and containment strategies (78).

Another critical challenge is the global disparity in vaccine and therapeutic access. Although smallpox vaccines such as Modified Vaccinia Ankara (MVA-BN) and antivirals like Tecovirimat show promise, their availability remains inequitable. Endemic regions, particularly in Central and West Africa, face severe shortages due to logistical and economic barriers (1). This lack of access not only exacerbates local outbreaks but also increases the risk of cross-border transmission, highlighting the need for globally coordinated efforts to ensure equitable resource distribution.

Community engagement and education are equally critical for managing MPXV outbreaks. Public awareness campaigns tailored to local contexts can empower communities to adopt preventive measures, recognize symptoms, and seek timely medical care. Such efforts should also address misinformation, which can undermine public trust and complicate outbreak responses (2). Integrating these strategies with surveillance systems and early diagnostic tools will improve outbreak detection and containment.

International collaboration remains essential for addressing the multifaceted challenges posed by MPXV. Coordinated efforts to harmonize diagnostic protocols, share epidemiological data, and accelerate vaccine and therapeutic development can strengthen global preparedness. The establishment of global frameworks, similar to those developed for COVID-19, can facilitate rapid response to emerging MPXV outbreaks (16). Furthermore, these collaborations should prioritize capacity building in endemic regions, ensuring sustainable improvements in healthcare infrastructure and outbreak management.

The resurgence of MPXV provides valuable insights into the broader dynamics of zoonotic disease emergence. Drivers such as deforestation, biodiversity loss, and climate change increase the frequency of spillover events, underscoring the need to address these root causes through One Health approaches. Strengthening global health systems to manage MPXV will not only mitigate its impact but also enhance resilience against future zoonotic threats.

## Conclusion

The re-emergence of MPXV serves as a critical reminder of the importance of sustained vigilance and coordinated global efforts in managing zoonotic diseases. Surveillance, vaccination, and public education must form the backbone of a comprehensive response, supported by equitable access to diagnostics and therapeutics. International collaboration is imperative for bridging resource gaps, harmonizing research efforts, and ensuring preparedness for future outbreaks.

By addressing the ecological, social, and immunological drivers of MPXV resurgence, the global health community has an opportunity to develop robust systems that mitigate the risks of zoonotic diseases. Failure to act decisively could result in the normalization of MPXV as a persistent global health challenge, with far-reaching consequences for vulnerable populations. Investing in research, infrastructure, and international partnerships will be critical in preventing future pandemics and ensuring global health security.

## Footnotes

^1^*APOBEC3* (apolipoprotein B mRNA-editing enzyme catalytic subunit 3) refers to a family of human cytidine deaminase enzymes. These host enzymes can deaminate cytosine (C) in single-stranded DNA to uracil (U), leading to characteristic **C → U (read as G → A)** hypermutations in viral genomes.
